# Insulin-Mimicking Bioactivities of Acylated Inositol Glycans in Several Mouse Models of Diabetes with or without Obesity

**DOI:** 10.1371/journal.pone.0100466

**Published:** 2014-06-27

**Authors:** Susumu Suzuki, Chitose Suzuki, Yoshinori Hinokio, Yasushi Ishigaki, Hideki Katagiri, Makoto Kanzaki, Viatcheslav N. Azev, Nilanjana Chakraborty, Marc d'Alarcao

**Affiliations:** 1 Department of Diabetes and Metabolism, Tohoku University Hospital, Sendai, Japan; 2 Diabetes Center, Ohta Nishinouchi Hospital, Koriyama, Japan; 3 Graduate School of Biomedical Engineering, Tohoku University, Sendai, Japan; 4 Department of Chemistry, Tufts University, Medford, Massachusetts, United States of America; 5 Department of Chemistry, San Jose State University, San Jose, California, United States of America; Northeast Ohio Medical University, United States of America

## Abstract

Insulin-mimetic species of low molecular weight are speculated to mediate some intracellular insulin actions. These inositol glycans, which are generated upon insulin stimulation from glycosylphosphatidylinositols, might control the activity of a multitude of insulin effector enzymes. Acylated inositol glycans (AIGs) are generated by cleavage of protein-free GPI precursors through the action of GPI-specific phospholipase C (GPI-PLC) and D (GPI-PLD). We synthesized AIGs (IG-1, IG-2, IG-13, IG-14, and IG-15) and then evaluated their insulin-mimicking bioactivities. IG-1 significantly stimulated glycogen synthesis and lipogenesis in 3T3-L1 adipocytes and rat isolated adipocytes dose-dependently. IG-2 significantly stimulated lipogenesis in rat isolated adipocytes dose-dependently. IG-15 also enhanced glycogen synthesis and lipogenesis in 3T3-L1 adipocytes. The administration of IG-1 decreased plasma glucose, increased glycogen content in liver and skeletal muscles and improved glucose tolerance in C57B6N mice with normal diets. The administration of IG-1 decreased plasma glucose in STZ-diabetic C57B6N mice. The treatment of IG-1 decreased plasma glucose, increased glycogen content in liver and skeletal muscles and improved glucose tolerance in C57B6N mice with high fat-diets and db/db mice. The long-term treatment of IG-1 decreased plasma glucose and reduced food intake and body weight in C57B6N mice with high fat-diets and ob/ob mice. Thus, IG-1 has insulin-mimicking bioactivities and improves glucose tolerance in mice models of diabetes with or without obesity.

## Introduction

The peptide hormone insulin regulates glucose homeostasis by modulating the action of a multitude of metabolic enzymes in the liver, adipose tissue, and skeletal muscle. Upon insulin receptor activation and phosphorylation of the insulin-receptor substrates (IRSs), insulin signaling branches into several pathways leading to glucose uptake, lipogenesis, glycogen synthesis, glycolysis, and other anabolic processes [1]. However, glucose metabolism by activation of glycogen synthase, mitochondrial pyruvate dehydrogenase, and other regulatory enzymes through protein dephosphorylation, remain incompletely explained by these models for insulin action.

The existence of an alternative pathway that involves an insulin-mimetic species of low molecular weight was proposed more than 30 years ago [2], [3]. Second messengers referred to as inositol phosphoglycans (IPGs) are generated upon insulin stimulation from glycosylphosphatidylinositol (GPI) and control the activity of a multitude of insulin effector enzymes [4]. The minimal structural requirements of IPGs for insulin-mimetic action have been debated. Two types of IPGs, the inositol phosphoglycans A (IPG-A) type and IPG-P type were suggested. The IPG-A type is generated by cleavage of GPI anchors through the action of GPI-specific phospholipase C (GPI-PLC) [5]. The insulin-mimetic effect of IPGs has been demonstrated in adipocytes [6], hepatocytes [7], and muscle cells [6]. In addition, IPGs are involved in mitogenic signaling *via* type I cytokine receptors [8], [9], suggesting a broader role of IPGs as second messengers in the signaling network of hormones and cytokines. However, the mechanistic details of IPG action remain largely unknown.

GPI-anchors with an inositol residue acylated with a fatty acid have been found in animals. A 2-palmitoyl group is added early in GPI biosynthesis, but is usually removed prior to translocation to the cell membrane [10]. These inositol-acylated GPI anchors or protein-free GPIs might serve as precursors of acylated inositol glycans (AIGs) through GPI-PLC or GPI-PLD. GPI-PLD and GPI-PLC also generate alkylacylphosphatidic or alkylacylglycerols, respectively from the hydrolysis of a truncated GPI. It has been reported that insulin activates GPI-PLC and GPI-PLD in adipocytes and hepatocytes [4], [6], [7], [11]. It was shown that GPI-PLD converts protein-free GPIs into AIG [11]. These AIG models other than lipidated GPI anchors, have not been described in the literature.

We prepared a series of AIGs, including IG-1 (2-amino-2-deoxy-α-D-glucopyranosyl-(1->6)-2-palmitoyl-D-*myo*-inositol), IG-2 (2-amino-2-deoxy-α-D-glucopyranosyl-(1->6)-2-palmitoyl-D-*myo*-inositol-1-phosphate), IG-13 (2-amino-2-deoxy-α-D-glucopyranosyl-(1->6)-1-palmitoyl-D-*myo*-inositol), IG-14 (2-amino-2-deoxy-α-D-glucopyranosyl-(1->3)-2-palmitoyl-D-*myo*-inositol), and IG-15 (2-amino-2-deoxy-α-D-glucopyranosyl-(1->3)-1-palmitoyl-D-*myo*-inositol) via chemical synthesis. The chemical structures of these AIGs are shown in Fig. S1. The details of these syntheses will be reported elsewhere. To test the possibility that AIGs can serve as mediators of insulin action, we evaluated their insulin-mimicking bioactivities.

## Methods and Materials

### Glycogen synthesis and lipogenesis in 3T3-L1 adipocytes

3T3-L1 fibroblasts were maintained in Dulbecco's modified Eagle's medium (DMEM) with 25 mM glucose plus 10% fetal bovine serum (FBS). Following 2 days at confluence, differentiation was initiated by the addition of DMEM containing 10% FBS, 167 nM insulin, 0.25 µmol/liter dexamethasone, and 0.5 mM isobutylmethylxanthine. Three days later, the medium was replaced with DMEM plus 10% FBS and 167 nM insulin. After 2 more days, the medium was switched to DMEM plus 10% fetal bovine serum. Adipocytes were used 6–14 days after completion of the differentiation protocol, when >90% of the cells expressed the adipocyte phenotype. Prior to experiments, cells were washed two times with DMEM medium containing 5 mM glucose, 0.2% bovine serum albumin, 25 mM Hepes (pH 7.4), 100 units/ml penicillin, 100 units/ml streptomycin and 0.29 mg/ml glutamine, and were incubated in the same medium for 2 h. Glycogen and lipid synthesis were measured in 24-well dishes. Following pretreatment with various concentrations of AIGs in DMSO for 30 min, cells were stimulated for 30 min in the absence and presence of 1–100 nM insulin. Then 1 µCi of [^14^C]-glucose (approximately 220 cpm/nmol) was added to all wells. After a 60-min incubation at 37°C, cells were washed three times on ice with PBS, and adipocytes were collected in 1 ml of distilled water. 400 µl of the cell suspension was added to 400 µl of PBS. The lipids were then extracted overnight with 5 ml of Betafluor (National Diagnostics), and glucose incorporation into lipid was measured by scintillation counting. 400 µl of the cell suspension was added to 600 µl of 50% KOH, and glycogen was precipitated and radioactivity was counted by a liquid scintillation counter. DNA content in 100 µl of the cell suspension was measured using the QIAGEN DNeasy kit, and counts per minutes were converted to picomoles glucose and normalized to the number of cell per dishes.

### Lipogenesis in native rat adipocytes

Isolated native rat adipocytes were prepared and lipogenesis was measured as previously [12]. Rat adipocytes were incubated with 6-[^3^H]-glucose (0.55 mM) and various concentrations of IG-1 and IG-2 for 1 hour. Incorporation of tritium into lipids was measured and is expressed as a percent of the maximal insulin response (%MIR).

### Animal studies

All experiments were approved by the Center for Laboratory Animal Research, Tohoku University (Permit Number: 21-Idou-149), and all experiments were conducted according to the Guidelines of the National Institutes of Health and the Tohoku University Guidelines for Animal Care and Use. Male C57BL/6 mice were housed individually and given free access to a standard diet (65% carbohydrate, 11% fat, and 24% protein) or a high-fat diet (Quick Fat; 60.2% carbohydrate, 15.3% fat, and 24.5% protein; Clea Japan, Tokyo, Japan), starting at 5 weeks of age, for 5 weeks. db/db and ob/ob mice also were housed individually and given free access to a standard diet. C57BL/6 mice at 5 weeks of age received a single dose of 75 µg/g body weight streptozotoxin (STZ). 7 days after injections, the mice, whose fasting plasma glucose concentrations were over 400 mg/dl, were used as STZ-diabetic mice.

### Glycogen contents of the liver and ground hind limb muscles

Glycogen was isolated from 30–50 mg of frozen liver and ground hind limb muscles by dissolving the tissue in 30% potassium hydroxide saturated with Na_2_SO_4_ for 30 min at 100°C, followed by ethanol precipitation. Glycogen content was determined by the phenol-sulfuric acid spectrophotometric method at 490 nm [13] and expressed as micrograms of glycogen per milligram of liver.

### Blood analysis

Blood glucose was assayed with Glucose Ace (Sanwa Kagaku Kenkyusho, Nagoya, Japan). Serum insulin and leptin were determined with ELISA kits (Morinaga Institute of Biological Science, Yokohama, Japan). Serum total adiponectin and tumor necrosis factor-α (TNF-α) concentrations were measured with an ELISA kit (Ohtsuka Pharmaceutical, Tokyo, Japan) and a TNF-α assay kit (Amersham Biosciences, Uppsala, Sweden), respectively. Serum total cholesterol, triglyceride, and FFA concentrations were determined with a Cholescolor liquid, Lipidos liquid (TOYOBO, Osaka, Japan), and NEFA C (Wako Pure Chemical, Osaka, Japan) kits, respectively.

### Glucose tolerance tests

Glucose tolerance was assessed with intraperitoneal glucose injection. Glucose tolerance tests were performed on fasted (16 h) mice. Mice were given intraperitoneal glucose (2 g/kg body wt), and blood glucose was assayed immediately before and at 15, 30, 60, and 120 min after administration. Area under the curve (AUC) was calculated by the trapezoid rule for the glucose tolerance curve using the XLSTAT add-in for Microsoft Excel software (Mindware Software).

### Intravenous administration of IG-1

Intravenous administration of IG-1 was performed on mice fed ad libitum. Mice received an injection of IG-1 or human regular insulin (1 units/kg body wt for normal diets; 2 units/Kg for high fat diets, db/db, and STZ-diabetic C57B/6N mice; Eli Lilly, Kobe, Japan) from tail veins, and blood glucose was assayed immediately before and at 30, 60, 90, 120, 180, 240 and 300 min after injection.

### Statistical Analysis

All data are expressed as mean±SEM. Normality was tested with the Kolmogorov-Smirnov test. When data were normally distributed, the statistical significance of differences was assessed by one-way or two-way ANOVA. Multiple experimental groups were compared by use of Tukey-Kramer post hoc test. In all analyses, values of P<0.05 were accepted as statistically significant.

## Results

### Insulin mimicking bioactivities of IG-1 in 3T3-L1 adipocytes and native rat adipocytes

**Figure 1:**
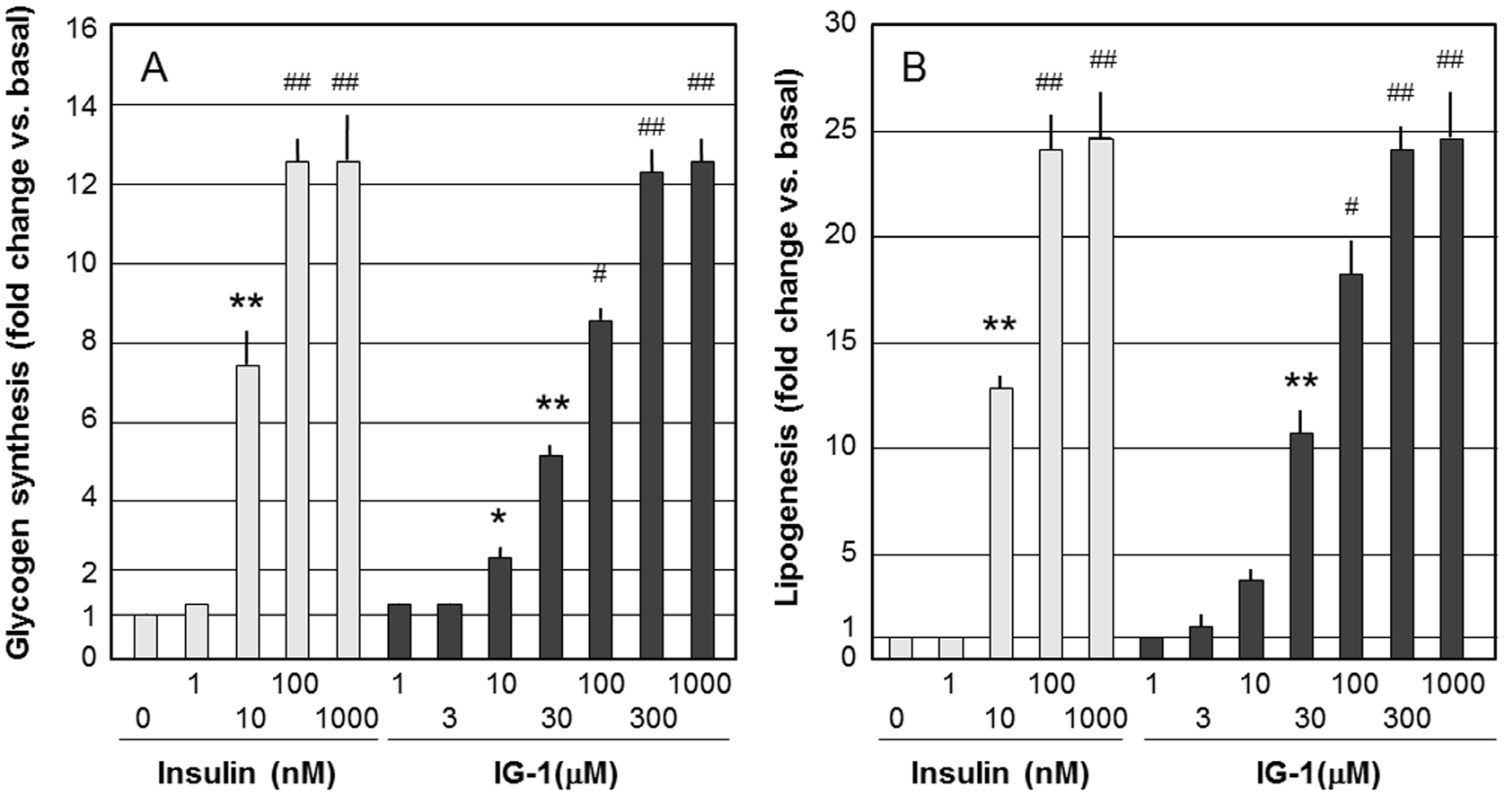
The effects of IG-1 on glycogen synthesis (A) and lipogenesis (B) in 3T3-L1 adipocytes. 3T3-L1 adipocytes were treated with various concentrations of IG-1 in DMSO or 1–1000 nM insulin for 30 min. Then 1 µCi of [^14^C]-glucose (approximately 220 cpm/nmol) was added and glycogen and lipid synthesis were measured as described in Methods and Materials. Values are expressed as fold change vs. basal. Graphs show the means±SEM of 3 independent experiments. *p<0.05, **p<0.01, ^#^p<0.001, ^##^p<0.0001 (vs. insulin 0 nM), assessed by one-way ANOVA followed by Tukey-Kramer post hoc test.

**Figure 2:**
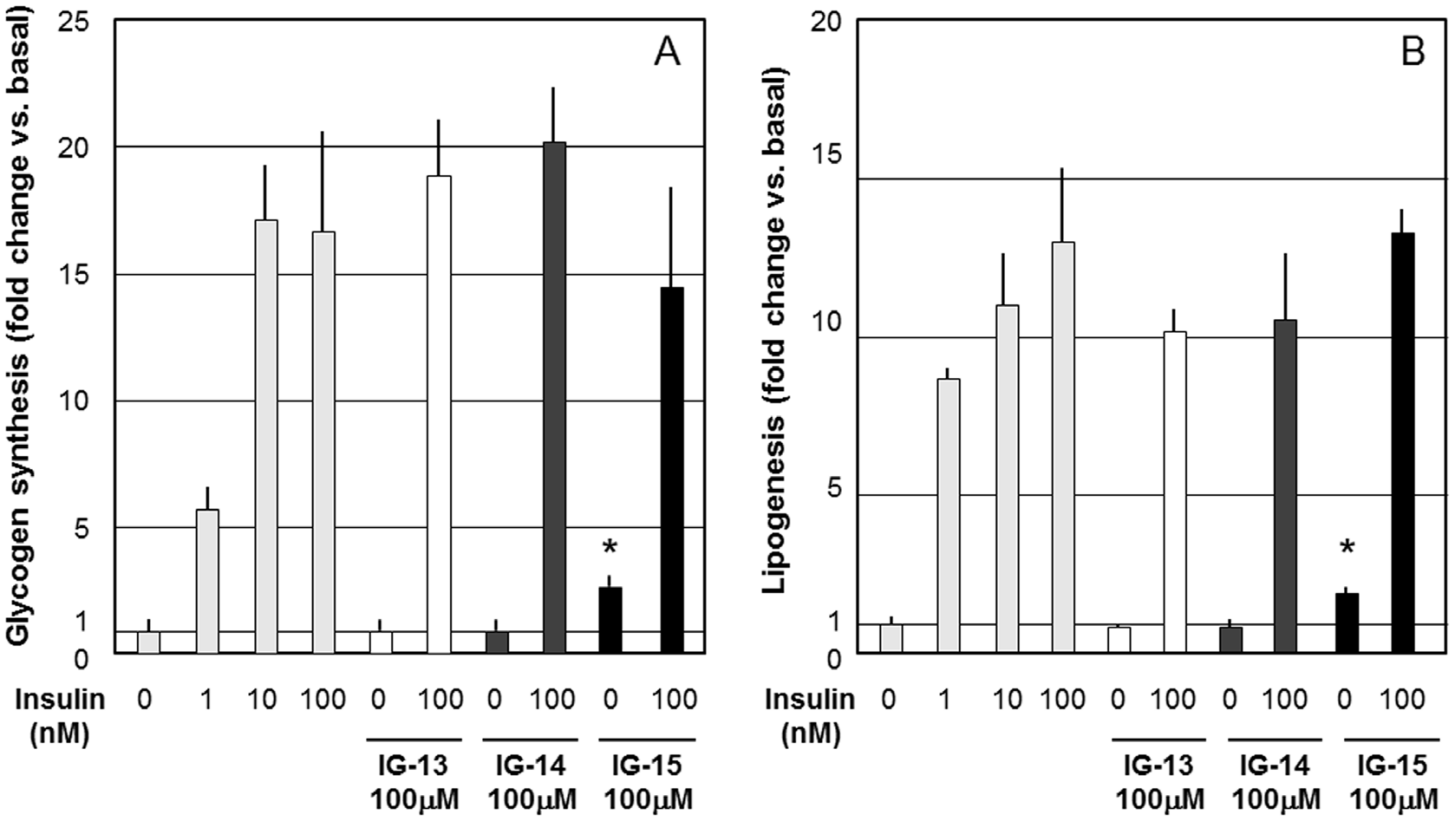
The effects of IG-13, IG-14 and IG-15 on glycogen synthesis (A) and lipogenesis (B) in 3T3-L1 adipocytes. 3T3-L1 adipocytes were treated with 1–100 nM insulin in the presence or absence of 100 µM IG-13, IG-14, or IG-15 in DMSO for 30 min. Then 1 µCi of [^14^C]-glucose (approximately 220 cpm/nmol) was added and glycogen and lipid synthesis were measured as described in Methods and Materials. Values are expressed as fold change vs. basal. Graphs show the means±SEM of 3 independent experiments. *p<0.05 (vs. insulin 0 nM), assessed by one-way ANOVA followed by Tukey-Kramer post hoc test.

IG-1 significantly stimulated glycogen synthesis and lipogenesis in 3T3-L1 adipocytes at the concentration of 10–1000 µM (Fig. 1A,B). IG-2 did not stimulate glycogen synthesis and lipogenesis in 3T3-L1 adipocytes (Fig. S2A,B). IG-1 and IG-2 did not stimulate glucose transport in 3T3-L1 adipocytes (data not shown). In contrast with insulin treatment, the treatment with IG-1 for 1–7 days did not stimulate DNA synthesis and proliferation of 3T3-L1 adipocytes (data not shown). IG-15 significantly stimulated glycogen synthesis and lipogenesis in 3T3-L1 adipocytes at the concentration of 100 µM (Fig. 2A,B). IG-1 and IG-2 significantly stimulated lipogenesis in native rat adipocytes in a dose-dependent manner (Fig. S3A,B).

### One shoot venous administration of IG-1 reduced plasma glucose and improved glucose tolerance in C57B6N mice fed normal diets

**Figure 3:**
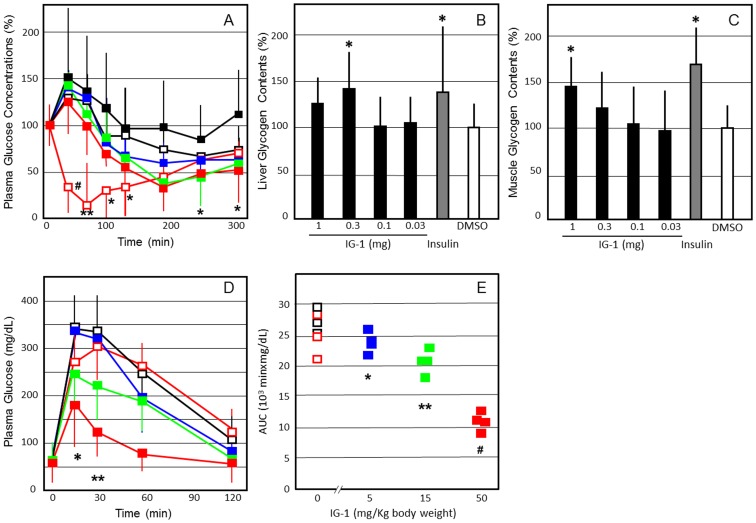
The effects of IG-1 on plasma glucose concentrations (A), glycogen contents in the liver (B) and skeletal muscles (C), and intraperitoneal GTT curve (D) and AUC values (E) after 7 days in the C57B/6N mice fed with normal diets. **A**. C57B/6N mice fed with normal diets received an injection of IG-1 (red square; 1 mg/day, green square; 0.3 mg/day, blue square; 0.1 mg/day, black square; 0.03 mg/day), DMSO (black open square) or human regular insulin (red open square; 1 units/kg body wt) from tail veins, and blood glucose was assayed immediately before and at 30, 60, 90, 120, 180, 240 and 300 min after injection. Values are expressed as % change from preadministraion of IG-1 of each group. **B**,**C**; C57B/6N mice fed with normal diets received an injection of 0.03–1 mg of IG-1, DMSO or human regular insulin (1 units/kg body wt) from tail veins, and glycogen contents of the liver and skeletal muscles were measured as described in Methods and Materials. Basal glycogen contents in the liver and skeletal muscles were 9.28±1.04 and 1.51±0.29 mg/g tissue, respectively. **D**,**E**; C57B/6N mice fed with normal diets (body weight 19.3±1.6 g) received intraperitoneal injection of IG-1 (0.1–1.0 mg/day), DMSO or human regular insulin (1 units/kg body wt) for 7 days. IG-1 doses per body weights were 51.8±4.3 mg/Kg (1 mg group),15.5±1.3 mg/Kg (0.3 mg) and 5.18±0.43 mg/Kg (0.1 mg). Overnight-fast mice were given intraperitoneal glucose (2 g/kg body wt), and blood glucose was assayed immediately before and at 15, 30, 60, and 120 min after administration. Values are expressed as AUC based on weighted means of all glucose measurements (t = 0, 15, 30, 60, 120 min). Similar representative results were obtained from 3 experiments, and the data are presented as means±SEM (n = 4). *p<0.05, **p<0.01, ^#^p<0.001 (vs. DMSO), assessed by one-way ANOVA followed by Tukey-Kramer post hoc test.

When 1 mg of IG-1 was administered intravenously to the C57B6N mice fed with normal diets, plasma glucose concentrations were significantly reduced from 180 min to 240 min, as compared with DMSO administration (Fig. 3A). On the next day after one-shoot intravenous injection, fasting plasma glucose levels showed no significant difference between the IG-1 and DMSO groups. However, glycogen content in mice liver and skeletal muscles was increased significantly (P<0.05) (Fig. 3B,C).

Daily administration of IG-1 for 7 days did not significantly affect food intake, body weights, fasting plasma glucose, insulin and triglyceride levels in the mice with normal diets (data not shown). However, glucose tolerance tests were performed on C57BL/6 mice fed with normal diets that had fasted overnight and had received peritoneal injection of 2 g glucose/kg body weights. Fasting plasma glucose levels showed no significant difference between the IG-1 and DMSO groups. However, after glucose load, the AUC levels were significantly lower at doses of 5.18 mg/Kg or higher in the mice injected with IG-1 (P<0.05) (Fig. 3D,E). Fasting insulin levels showed no significant difference between the IG-1 and DMSO groups, however, plasma insulin levels were significantly reduced in the mice injected with IG-1 after 30 minutes (data not shown) (P<0.05).

### The treatment with IG-1 decreased plasma glucose, improved glucose tolerance, and reduced food intake and body weight in C57B6N mice fed with high fat diets and db/db mice, models of obese diabetes mellitus

**Figure 4:**
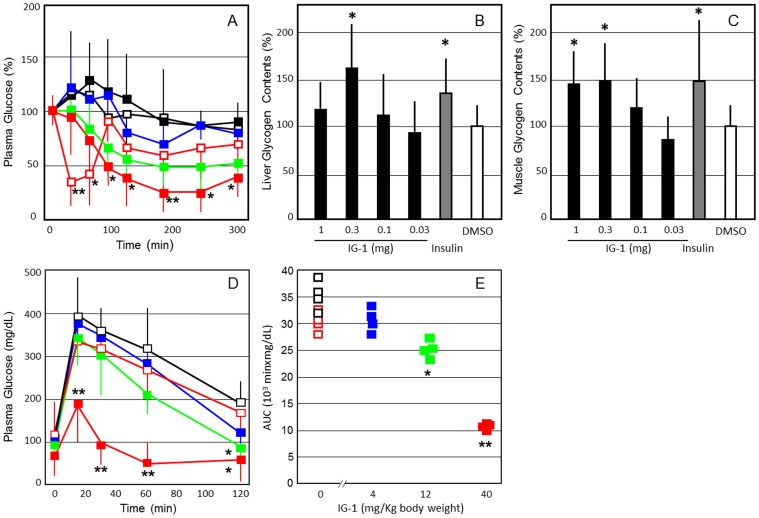
The effects of IG-1 on plasma glucose concentrations (A), glycogen contents in the liver (B) and skeletal muscles (C), and intraperitoneal GTT curve (D) and AUC values (E) after 7 days in the C57B/6N mice fed with high fat diets. **A**; Mice fed with high fat diets received an injection of IG-1 (red square; 1 mg/day, green square; 0.3 mg/day, blue square; 0.1 mg/day, black square; 0.03 mg/day), DMSO (black open square) or human regular insulin (red open square; 1 units/kg body wt) from tail veins, and blood glucose was assayed immediately before and at 30, 60, 90, 120, 180, 240 and 300 min after injection. Values are expressed as % change from preadministraion of IG-1 of each group. **B**,**C**; Glycogen contents of the liver and skeletal muscles were measured as described in Methods and Materials. Basal glycogen contents in the liver and skeletal muscles were 8.42±1.46 and 1.33±0.29 mg/g tissue, respectively. **D**,**E**; C57B/6N mice fed with high fat diets (body weight 24.3±2.1 g) received intraperitoneal injection of IG-1 (0.1–1.0 mg/day), DMSO or human regular insulin (2 units/kg body wt) for 7 days. IG-1 doses per body weights were 41.2±3.5 mg/Kg (1 mg group), 12.3±1.1 mg/Kg (0.3 mg) and 4.12±0.35 mg/Kg (0.1 mg). Overnight-fast mice were given intraperitoneal glucose (2 g/kg body wt), and blood glucose was assayed immediately before and at 15, 30, 60, and 120 min after administration. Values are expressed as AUC based on weighted means of all glucose measurements (t = 0, 15, 30, 60 and 120 min). Similar representative results were obtained from 3 experiments, and the data are presented as means±SEM (n = 4). *p<0.05, **p<0.01, ^#^p<0.001 (vs. DMSO), assessed by one-way ANOVA followed by Tukey-Kramer post hoc test.

One shoot venous administration of IG-1 (1 mg) significantly decreased plasma glucose concentrations from 90 min to 300 min in the C57B6N mice fed with high fat diets (Fig. 4A), as compared with DMSO treatment. On the day after one-shoot intravenous injection, fasting plasma glucose levels showed no significant difference between the IG-1 and DMSO groups (data not shown). However, glycogen content in mice liver and skeletal muscles was increased significantly in IG-1 treated mice (P<0.05) (Fig. 4B,C).

When IG-1 (40 µg/g body weight) was administrated to the C57B6N mice fed high fat-diets for 7 days, food intake of the treated mice was significantly lower than those of the control mice (Table 1). In addition, body weights were also significantly decreased in the IG-1-treated mice as compared with the control mice. Fasting plasma glucose, insulin and triglyceride levels were markedly decreased in the IG-1 treated mice than the control mice at the 7 days after IG-1 injection.

**Table 1:** Metabolic parameters in the C57B/6N mice on high fat diets after the IG-1 treatment for 7 days.

	DMSO	IG-1 (40 µg/g body weight)	p value
Body weight (g)	34.7±1.9	32.1±3.6	p<0.05
Food intake (g/day)	4.04±0.81	2.93±0.73	p<0.01
Fasting plasma glucose (mg/dL)	104.2±30.6	62.8±11.8	p<0.01
Fasting plasma insulin (ng/mL)	1.13±0.59	0.44±0.38	p<0.01
Total cholesterol (mg/dL)	201.3±23.9	177.9±47.1	NS
Triglyceride (mg/dL)	34.7±8.0	26.2±8.2	p<0.05
NEFA (mEq/L)	1.12±0.4	0.78±0.33	p<0.05
Adiponectin (µg/mL)	49.3±7.3	50.4±9.2	NS
TNF-α (pg/mL)	32.7±5.7	33.7±8.8	NS
Leptin (ng/mL)	33.7±14.3	17.3±10.2	p<0.05

Mean ± SEM (n = 6).

After 7 days of IG-1 treatment, glucose tolerance tests were performed on C57BL/6 mice with high fat diets. Fasting plasma glucose levels showed no significant difference between the IG-1 and DMSO groups in mice with high fat-diets. However, after glucose load, the AUC levels were significantly lower at doses of 12.3 mg/Kg or higher in the IG-1-treated mice with high fat diets (Fig. 4D,E) (P<0.05). Fasting insulin levels showed no significant difference between the IG-1 and DMSO groups, however, plasma insulin levels were significantly reduced in the mice injected with IG-1 (41.2±3.5 mg/Kg) after 30 minutes in those two mice (data not shown) (P<0.05). These findings indicate that IG-1 exerted therapeutic effects on diabetic animal models of diet-induced obesity.

**Figure 5:**
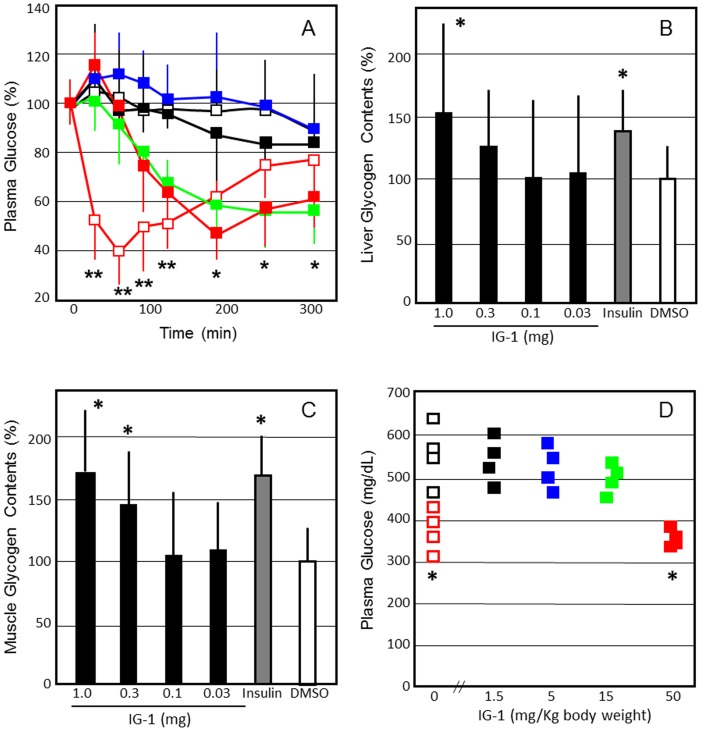
The effects of IG-1 on plasma glucose concentrations (A), glycogen contents in the liver (B) and skeletal muscles (C), and fasting plasma glucose concentrations after 7 days in the STZ-diabetic C57B/6N mice. **A**; STZ-diabetic C57B/6N mice received an injection of IG-1 (red square; 1 mg/day, green square; 0.3 mg/day, blue square; 0.1 mg/day, black square; 0.03 mg/day), DMSO (black open square) or human regular insulin (red open square; 2 units/Kg) from tail veins, and blood glucose was assayed immediately before and at 30, 60, 90, 120, 180, 240 and 300 min after injection. Values are expressed as % change from preadministraion of IG-1 of each group. **B**,**C**; Glycogen contents of the liver and skeletal muscles were measured as described in Methods and Materials. Basal glycogen contents in the liver and skeletal muscles were 7.47±1.29 and 1.34±0.27 mg/g tissue, respectively. **D**; STZ-diabetic C57B/6N mice (body weight 19.4±1.4 g) received intraperitoneal injection of 0.03–1 mg of IG-1 in DMSO or human regular insulin (2 units/kg body wt) for 7 days. IG-1 doses per body weights were 51.5±3.7 mg/Kg (1 mg group), 15.5±1.1 mg/Kg (0.3 mg), 5.15±0.37 mg/Kg (0.1 mg) and 1.55±0.11 mg/Kg (0.03 mg). After overnight fasting, fasting plasma glucose was measured. Similar representative results were obtained from 3 experiments, and the data are presented as means±SEM (n = 4). *p<0.05, **p<0.01, ^#^p<0.001, (vs. DMSO), assessed by one-way ANOVA followed by Tukey-Kramer post hoc test.

Intravenous administration of IG-1 (1 mg) significantly reduced plasma glucose concentrations in streptozotocin (STZ)-diabetic mice in 180–300 min after administration (Fig. 5A). Intravenous administration of IG-1 significantly increased glycogen contents in mice liver and skeletal muscles of the STZ-diabetic mice (P<0.05) (Fig. 5B,C). When IG-1 was administrated to the STZ-diabetic mice for 7 days, fasting plasma glucose was significantly lower at a dose of 51.5±3.7 mg/Kg in the IG-1-treated STZ-diabetic mice than in the untreated STZ-diabetic mice (P<0.05) (Fig. 5D). These findings indicate that IG-1 exerted therapeutic effects in an animal model of insulin-deficient type 1 diabetes.

**Figure 6:**
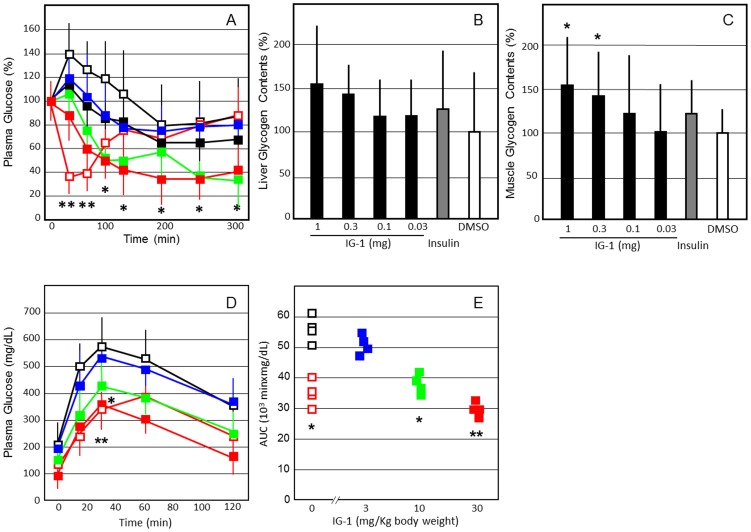
The effects of IG-1 on plasma glucose (A), glycogen contents in the liver (B) and skeletal muscles (C), and intraperitoneal GTT curve (D) and AUC values (E) after 7 days in the db/db obese diabetic mice. **A**; Overnight-fasting db/db mice received an injection of IG-1 (red square; 1 mg/day, green square; 0.3 mg/day. blue square; 0.1 mg/day, black square; 0.03 mg/day), DMSO (black open square) or human regular insulin (red open square; 2 units/kg body wt) from tail veins, and blood glucose was assayed immediately before and at 30, 60, 90, 120, 180, 240 and 300 min after injection. Values are expressed as % change from preadministraion of IG-1 of each group. **B**,**C**; Glycogen contents of the liver and skeletal muscles were measured as described in Methods and Materials. Basal glycogen contents in the liver and skeletal muscles were 8.24±4.14 and 1.33±0.29 mg/g tissue, respectively. **D**,**E**; The db/db mice (body weight 30.1±4.0 g) received intraperitoneal injection of IG-1 (0.1–1.0 mg/day), DMSO or human regular insulin (2 units/kg body wt) for 7 days. IG-1 doses per body weights were 33.2±4.4 mg/Kg (1 mg group), 10.0±1.3 mg/Kg (0.3 mg) and 3.32±0.44 mg/Kg (0.1 mg). Overnight-fast mice were given intraperitoneal glucose (2 g/kg body wt), and blood glucose was assayed immediately before and at 15, 30, 60, and 120 min after administration. Values are expressed as AUC based on weighted means of all glucose measurements (t = 0, 15, 30, 60 and 120 min). Similar representative results were obtained from 3 experiments, and the data are presented as means±SEM (n = 4). *p<0.05, **p<0.01 (vs. DMSO), assessed by two-way ANOVA followed by Tukey-Kramer post hoc test.

One shoot venous administration of IG-1 (1 mg) significantly decreased plasma glucose concentrations from 90 min to 240 min in the db/db mice, obese diabetic animal models (Fig. 6A), as compared with DMSO treatment. On the day after one-shoot intravenous injection, fasting plasma glucose levels showed no significant difference between the IG-1 and DMSO groups. However, glycogen contents in skeletal muscles were increased significantly (P<0.05) (Fig. 6C).

When IG-1 was administrated to the db/db mice for 7 days, food intake of the treated mice was significantly lower than those of the control mice (data not shown) (P<0.05). In addition, body weights were also significantly decreased in the IG-1-treated mice as compared with the control mice (data not shown) (P<0.05). Fasting plasma glucose, insulin and triglyceride levels were markedly lower in the IG-1-treated mice than in the control mice at the 7 days after IG-1 injection (data not shown) (P<0.05).

After 7 days of IG-1 treatment, glucose tolerance tests were performed on db/db mice. Fasting plasma glucose levels showed no significant difference between the IG-1 and DMSO groups in db/db mice. However, after glucose load, the AUC levels were significantly lower at doses of 10.0 mg/Kg or higher in IG-1-treated db/db mice (P<0.05) (Fig. 6D,E). Fasting insulin levels showed no significant difference between the IG-1 and DMSO groups, however, plasma insulin levels at 30 minutes after glucose load were significantly lower in the mice injected with IG-1 at doses of 33.2±4.4 mg/Kg, (data not shown) (P<0.05).

IG-1 treatment for 28 days reduced food intake and body weight in the ob/ob mice, a genetic obese animal model (Table 2). IG-1 treatment also decreased fasting plasma glucose, insulin, triglyceride and free fatty acids levels in the ob/ob mice (Table 2). In addition IG-1 treatment increased serum adiponectin and decreased serum TNF-α in the ob/ob mice (Table 2).

**Table 2:** Metabolic parameters in the ob/ob obese mice after the IG-1 treatment for 28 days.

	DMSO	IG-1 (40 µg/g body weight)	p value
Body weight (g)	47.9±8.1	40.1±6.1	p<0.01
Food intake (g/day)	5.32±1.71	4.53±1.45	p<0.05
Fasting plasma glucose (mg/dL)	89.8±20.7	66.1±15.8	p<0.01
Fasting plasma insulin (ng/mL)	27.6±4.0	16.3±8.1	p<0.01
Total cholesterol (mg/dL)	185.6±32.1	172.4±26.4	NS
Triglyceride (mg/dL)	42.2±8.0	31.2±10.7	p<0.05
NEFA (mEq/L)	1.45±0.81	0.88±0.74	p<0.05
Adiponectin (mg/mL)	34.3±6.8	47.6±9.0	p<0.05
TNF-α (pg/mL)	49.7±8.3	34.7±12.8	NS

Mean ± SEM (n = 6).

Administration of IG-1 for 7 days significantly improved glucose tolerance dose-dependently at doses of in 5.18 mg/Kg or higher in the C57B/6N mice on normal diets (Fig. 3E), 12.3 mg/Kg or higher in the C57B/6N mice on high fat diets (Fig. 4E) and 10.0 mg/Kg or higher in db/db mice (Fig. 6E). Thus, long-term treatment with IG-1 improves glucose tolerance in normal mice and in mice models of type 2 diabetes, obesity and metabolic syndrome. The treatment with IG-1 for 7 or 28 days reduces daily food intakes, body weights, and fasting plasma concentrations of glucose, insulin, triglyceride and free fatty acids, and hepatic triglyceride contents in mice models of type 2 diabetes, obesity and metabolic syndrome (table 1 and 2).

## Discussion

Some intracellular insulin actions might be mediated by insulin-mimetic species of low molecular weight, such as IPGs, generated from the hydrolysis of GPI. Analysis of isolated insulin-mimetic IPGs from insulin sensitive mammalian tissue or lower eukaryotes revealed two structurally and functionally distinctive classes of IPGs [14]. Type-A IPGs consist of *myo*-inositol and D-glucosamine, whereas type-P IPGs contains 3-*O*-methyl-D-*chiro*-inositol and D-galactosamine [15]–[17]. IPG-As mimic the lipogenic activity of insulin in adipose tissue and inhibit cAMP-dependent protein kinase [14]. IPG-Ps mimic the glycogenic effect of insulin on muscle and liver and stimulate pyruvate dehydrogenase phosphatase [14]–[16]. While the composition of type-A IPGs implies a structural relationship to the GPI glycolipids, in contrast, the origin of IPG-P messengers is unclear. The similarity between IPG-A and GPI glycans is supported by the fact that antibodies raised against GPI-anchors cross-react with IPG preparations [18]. Moreover, insulin-stimulated glycogen synthesis in K562 cells requires the biosynthesis of GPI anchors [19]. GPI-anchored proteins are found in lipid rafts on virtually all eukaryotic cell surfaces [20]. They are particularly abundant on the surface of protozoan parasites. GPIs anchor proteins to the outer leaflet of the plasma membrane or occur in free from. The GPI core structure consists of EtN-P-6-Man-α1,2-Man-α1,6-Man-α1,4-GlcN-α1,6-*myo*-D-inositol-1-P-lipid that can be decorated by many different species- and tissue-specific modifications [21]. Lipolytically and proteolytically degraded GPI-anchored proteins from parasites [22] and yeast [23] have been shown to regulate glucose homeostasis in the rodent animal model.

A number of active IPG-As carry a 1,2-cyclic phosphate at *myo*-inositol [24]–[29]. It has been suggested that IPG-As are generated by cleavage of GPI anchors through the action of GPI-PLC [30], [31]. However, the gene for mammalian GPI-PLC has never been identified. The details of both generation of IPGs and IPG downstream signaling remain unclear. Despite intensive efforts using synthesized IPGs, there is no agreement on the chemical composition of an active IPG-A molecule. The GPI core glycan pseudopentasaccharide without any modifications was shown not to have biological activity [32]. It was recently reported that some synthetic IPGs lack insulin-mimetic activity [33].

So, we speculated that another insulin-mimicking variant of IPGs might be the AIGs that are generated from protein-free GPI anchors through the action of a GPI-PLD and GPI-PLC. AIG have ester-linked fatty acid at the C2 hydroxyl of inositol. Inositol acylation occurs during GPI biosynthesis. The presence of this lipid modification makes the GPI anchor inherently resistant to the action of bacterial PI-PLC [34].

The gene encoding GPI-PLC, which cleaves IPG or AIG from GPI, has still not been identified in mammalian and human genomic surveys. In contrast, accumulating evidence suggests that GPI-PLD (GPLD1) has an important role in insulin signaling and pathogenesis of insulin resistance and diabetes. Gene structure and tissue expression of GPLD1 has been well elucidated [35]. GPI-PLD is abundant in mammalian serum [36]. The source of the circulating enzyme is liver and pancreatic β-cells [37]–[39]. GPI-PLD expressed in pancreatic islet is regulated by glucose and insulin [37]. Glucose stimulates both GPI-PLD and insulin secretion from beta TC3 cells [38]. However, GPI-PLD secretion differs from insulin secretion by a higher rate of basal release and a lower magnitude of response to secretagogues [39].

Serum level of GPI-PLD was increased in human and rodent type 1 diabetes, but it was reduced by treatment with insulin [40], [41]. It was reported that insulin induced GPI hydrolysis by GPI-PLD [42]. Deeg et al reported that insulin resistance is associated with increased serum levels of GPI-PLD [43]. GPLD1 was reported to contribute to low fat diet-induced improvement of insulin sensitivity, but not low-carbohydrate diet-induced insulin sensitivity [44]. GPI-PLD is reported to contribute the pathogenesis of insulin-resistant pre-eclamsia through the association with IPG [45]. Grass pea (Lathyrus sativus) seeds have been used in some traditional medicines to ameliorate diabetic symptoms. It was reported that IPG generated from Lathyrus sativus seeds by hydrolysis with GPI-PLD, possesses insulin-mimetic activities [46]. GPI-PLD associated with HDL through the interaction with apoA-1, and apoA-IV [47], [48]. GPI-PLD was reported to contribute to triglyceride metabolism [49]. Statin regulated serum level of GPI-PLD [50]. GPI-PLD was reported to contribute to lipid metabolism in liver and pathogenesis of nonalcoholic fatty liver disease [51].

The work presented here aims at clarifying the active structure of GPI precursor-derived insulin-mimetic IPGs using synthetic AIGs. We investigated the insulin-mimetic potential of five synthetic AIGs in primary murine adipocytes, 3T3-L1 adipocytes, as well as type 1 and type 2 diabetic mice. IG-1 has insulin-mimicking bioactivities in primary murine adipocytes, 3T3-L1 adipocytes, as well as type 1 and type 2 diabetic mice and genetic obese mice. In contrast, IG-2 only stimulated lipogenesis in primary murine adipocytes, but not in 3T3-L1 adipocytes. IG-1 and IG-2 are expected to be generated from the hydrolysis of a protein-free GPI precursor by GPI-PLD and GPI-PLC, respectively. In contrast, less bioactive IG-15 with a 1-palmitoyl-group and alpha 1,3 linkage might not to be generated from GPI precursor. It has been reported that a minor fatty acid constituent found at the 2-position of myo-inositol in mammalian GPI is myristate [52]. We synthesized IG-21 (D-glucosamine-(α1,6)-(2-myristoyl)-D-myo-inositol) and other short-chain fatty acid (C2 to C12)-linked inositol glycans species and checked insulin-mimic bioactivities in 3T3-L1 adipocytes. However, we could not detect insulin-mimic bioactivities of those lipophilic inositol glycans (data not shown), suggesting that palmitoylation at the 2-position of myo-inositol might be critical for insulin-mimic bioactivities of lipophilic inositol glycans. The minimal active AIG structure remains elusive and new synthetic materials will be required for the study of insulin-mimetic AIGs.

Our results strongly support the notion that GPI derived AIGs have insulin-mimetic activity. These exciting results support the hypothesis that these truncated GPI products, IG-1 or IG-2 may be involved in insulin signaling in muscle, fat, and liver, as well as in body glucose and lipid homeostasis. This study confirmed that IG-1 has insulin-mimicking bioactivities and improves glucose tolerance in several mice models of diabetes with or without obesity, such as male C57BL/6 mice fed normal diets (normal model), mice fed high fat diets (model of diabetes with mild obesity), db/db mice (genetic model of diabetes with severe obesity), ob/ob mice (genetic model of severe obesity), STZ-diabetic mice (model of lean insulin-deficient diabetes), respectively. Our preliminary study demonstrated that IG-1 inhibited glucose-induced insulin secretion in Min-6 insulinoma cells (data not shown). Inhibition of insulin secretion by IG-1 may be a mechanism for turning off insulin action. Therefore, AIGs would be expected as useful tools in the treatment of diabetes, obesity and metabolic syndrome.

It is very interesting that long-term treatment with IG-1 reduced food intake and body weight, and increased adiponectin concentrations in diabetic obese mice. Anabolic actions of IG-1 might be mediated through the neural network from liver and adipose tissues to brain, resulting in decreased appetite and body adiposity, as well as increased adiponectin concentrations [53]. Another possibility is that IG-1 might move into the brain and regulate appetite as an anabolic signaling factor. IG-1 might affect adipose tissues as an anabolic signaling factor and regulate adiponectin secretion. Further investigation is needed to elucidate a possible role of IG-1 on appetite regulation.

We need further investigation to identify the insulin-sensitive GPI precursor and elucidate the molecular mechanism of GPI-PLD activation by insulin to generate IG-1 and phosphatidic acids. Further investigation is required to identify the down-stream signaling molecules activated by IG-1. We will clarify the extent of interaction between IG-1 and PAs on metabolic signaling of insulin. A clinical study should be conducted to elucidate the role of IG-1 in insulin resistance in type 2 diabetes, obesity and metabolic syndrome.

## Supporting Information

Figure S1 **Chemical structures of synthetic acylated inositol glycans.**
(ZIP)

Figure S2 **The effects of IG-1, IG-2, IG-13, IG-14 and IG-15 on lipogenesis in native rat adipocytes.** A: Rat adipocytes were incubated with 6-[^3^H]-glucose (0.55 mM) and various concentrations of IG-1 and IG-2 for 1 hour. Incorporation of tritium into lipids was measured and is expressed as a percent of the maximal insulin response (%MIR). Red circles: IG-1 (0–60 µM); black squares: IG-2 (0–60 µM). Each data point is the average of at least five replicates. Error bars represent ±1 SD. B: Rat adipocytes were incubated with 6-[^3^H]-glucose (0.55 mM) and 40 µM of IG-1, IG-2, IG-13, IG-14, or IG-15 for 1 hour. Incorporation of tritium into lipids was measured and is expressed as a percent of the maximal insulin response (%MIR). Each data point is the average of at least five replicates. Error bars represent ±1 SD.
(ZIP)

Figure S3 **IG-2 did not stimulated glycogen synthesis and lipogenesis in 3T3-L1 adipocytes.** 3T3-L1 adipocytes were treated with various concentrations of IG-2 in DMSO or 1–100 nM insulin for 30 min. Then 1 µCi of [^14^C]-glucose (approximately 220 cpm/nmol) was added and glycogen and lipid synthesis were measured as described in Methods and Materials. Values are expressed as fold change vs. basal. Graphs show the means±SEM of 3 independent experiments.
(ZIP)

## References

[1] Boura-Halfon S, Zick Y (2009) Phosphorylation of IRS proteins, insulin action, and insulin resistance. Am J Physiol Endocrinol Metab 296 4: E581–91. 18728222 10.1152/ajpendo.90437.2008

[2] Larner J, Galasko G, Cheng K, DePaoli-Roach AA, Huang L, et al (1979) Generation by insulin of a chemical mediator that controls protein phosphorylation and dephosphorylation. Science 206: 1408–1410. 228395 10.1126/science.228395

[3] Saltiel AR, Fox JA, Sherline P, Cuatrecasas P (1986) Insulin-stimulated hydrolysis of a novel glycolipid generates modulators of c-AMP phosphodiesterase. Science 233: 967–972. 3016898 10.1126/science.3016898

[4] Jones DR, Varela-Nieto I (1998) The role of glycosylphosphatidylinositol in signal transduction. Int J Biochem Cell Biol 30: 313–326. 9611774 10.1016/s1357-2725(97)00144-1

[5] Kunjara S, Wang DY, Greenbaum AL, McLean P, Kurtz A, et al (1999) Inositol phosphoglycans in diabetes and obesity: urinary levels of IPG A-type and IPG P-type, and relationship to pathophysiological changes. Mol Genet Metab 68 4: 488–502. 10607479 10.1006/mgme.1999.2936

[6] Kessler A, Muller G, Wied S, Crecelius A, Eckel J (1998) Signaling pathways of an insulin-mimetic phosphoinositolglycan peptide in muscle and adipose tissue. Biochem J 330: 277–286. 9461521 10.1042/bj3300277 PMC1219138

[7] Larner J, Price JD, Heimark D, Smith L, Rule G, et al (2003) Isolation, structure, synthesis, and bioactivity of a novel putative insulin mediator. A galactosamine chiro-inositol pseudo-disaccharide Mn^2+^ chelate with insulin-like activity. J Med Chem 46: 3283–3291. 12852758 10.1021/jm030071j

[8] Merida I, Pratt JC, Gaulton GN (1990) Regulation of interleukin 2-dependent growth responses by glycosylphosphatidylinositol molecules. Proc Natl Acad Sci USA 87: 9421–9425. 2147515 10.1073/pnas.87.23.9421 PMC55177

[9] Boudot C, Kadri Z, Petitfrere E, Lambert E, Chretien S, et al (2002) Phosphatidylinositol 3-kinase regulates glycosylphosphatidylinositol hydrolysis through PLC-γ2 activation in erythropoietin-stimulated cells. Cell Signaling 14: 869–878. 10.1016/s0898-6568(02)00036-0 12135708

[10] Ferguson MAJ (1999) The structure, biosynthesis and functions of glycosylphosphatidylinositol anchors, and the contributions of trypanosome research. Journal of Cell Science 112 17: 2799–809. 10444375 10.1242/jcs.112.17.2799

[11] Jones DR, Avila MA, Sanz C, Varela-Nieto I (1997) Glycosyl-phosphatidylinositol-phospholipase type D: A possible candidate for the generation of second messengers. Biochem Biophys Res Commun 233: 432–427. 9144552 10.1006/bbrc.1997.6475

[12] Turner DI, Chakraborty N, d'Alarcao M (2005) A fluorescent inositol phosphate glycan stimulates lipogenesis in rat adipocytes by extracellular activation alone. Bioorg Med Chem Lett 15: 2023–2025. 15808461 10.1016/j.bmcl.2005.02.061

[13] Shashkin PN, Wasner HK, Ortmeyer HK, Hansen BC (2001) Prostaglandylinositol cyclic phosphate (cPIP): a novel second messenger of insulin action. Comparative analysis of two kinds of ‘insulin mediators’. Diab Metab Res Rev 17 4: 273–284. 10.1002/dmrr.218 11544611

[14] Frick W, Bauer A, Bauer J, Wied S, Muller G (1998) Structure-activity relationship of synthetic phosphoinositolglycans mimicking metabolic insulin action. Biochemistry 37 38: 13421–13436. 9748349 10.1021/bi9806201

[15] Jones DR, Varela-Nieto I (1999) Diabetes and the role of inositol-containing lipids in insulin signaling. Mol Med 5 8: 505–514. 10501653 PMC2230454

[16] Larner J, Price JD, Heimark D, Smith L, Rule G, et al (2003) Isolation, structure, synthesis, and bioactivity of a novel putative insulin mediator. A galactosamine chiro-inositol pseudo-disaccharide Mn^2+^ chelate with insulin-like activity. J Med Chem 46 15: 3283–91. 12852758 10.1021/jm030071j

[17] Chakraborty N, d'Alarcao M (2005) An anionic inositol phosphate glycan pseudotetrasaccharide exhibits high insulin-mimetic activity in rat adipocytes. Bioorg Med Chem 13 24: 6732–6741. 16115771 10.1016/j.bmc.2005.07.020

[18] Alvarez JF, Sanchez-Arias JA, Guadano A, Estevez F, Varela I, et al (1991) Transport in isolated rat hepatocytes of the phospho-oligosaccharide that mimics insulin action. Effects of adrenalectomy and glucocorticoid treatment. Bioch J 274 2: 369–374. 10.1042/bj2740369 PMC1150146 2006906

[19] Larner J, Huang LC (1999) Identification of a novel inositol glycan signaling pathway with significant therapeutic relevance to insulin resistance: an insulin signaling model using both tyrosine kinase and G- proteins. Diabetes Rev 7 3: 217–231.

[20] Muller G, Jung C, Frick W, Bandlow W, Kramer W (2002) Interaction of phosphatidylinositolglycan(-peptides) with plasma membrane lipid rafts triggers insulin-mimetic signaling in rat adipocytes. Arch Biochem Biophy 408 1: 7–16. 10.1016/s0003-9861(02)00450-2 12485598

[21] Brewis IA, Ferguson MA, Mehlert A, Turner AJ, Hooper NM (1995) Structures of the glycosyl-phosphatidylinositol anchors of porcine and human renal membrane dipeptidase. Comprehensive structural studies on the porcine anchor and interspecies comparison of the glycan core structures. J Biol Chem 270: 22946–22956. 7559431 10.1074/jbc.270.39.22946

[22] Misek DE, Saltiel AR (1992) An inositol phosphate glycan derived from a Trypanosoma brucei glycosyl-phosphatidylinositol mimics some of the metabolic actions of insulin. J Biol Chem 267 23: 16266–73. 1322896

[23] Muller G, Wied S, Crecelius A, Kessler A, Eckel J (1997) Phosphoinositolglycan-peptides from yeast potently induce metabolic insulin actions in isolated rat adipocytes, cardiomyocytes, and diaphragms. Endocrinology 138 8: 3459–75. 9231801 10.1210/endo.138.8.5308

[24] Muller G, Frick W (1999) Signalling via caveolin: involvement in the cross-talk between phosphoinositolglycans and insulin. Cell Molec Life Sci 56 11–12: 945–970. 11212327 10.1007/s000180050485 PMC11146787

[25] Shashkin PN, Shashkina EF, Fernqvist Forbes E, Zhou YP, Grill V, et al (1997) Insulin mediators in man: Effects of glucose ingestion and insulin resistance. Diabetologia 40 5: 557–563. 9165224 10.1007/s001250050715

[26] Ferguson MAJ (1999) The structure, biosynthesis and functions of glycosylphosphatidylinositol anchors, and the contributions of trypanosome research. J Cell Sci 112 17: 2799–2809. 10444375 10.1242/jcs.112.17.2799

[27] Suzuki S, Taneda Y, Hirai S, Satoh Y, Toyota T (1993) Insulin stimulates hydrolysis of plasmanylinositol-glycan and phosphatidylinositol-glycan in rat adipocytes. Insulin-induced generation of inositol glycan, alkylacylglycerol, and diacylglycerol. Diabetes 42 7: 988–994. 8390376 10.2337/diab.42.7.988

[28] Suzuki S, Taneda Y, Hirai S, Yamamoto-Honda R, Toyota T (1992) Mutated insulin receptor Val996 reduces insulin-dependent generation of inositol glycan and diacylglycerol. Diabetes 41 11: 1373–1379. 1327926 10.2337/diab.41.11.1373

[29] Sleight S, Wilson BA, Heimark DB, Larner J (2002) G(q/11) is involved in insulin-stimulated inositol phosphoglycan putative mediator generation in rat liver membranes: co-localization of G(q/11) with the insulin receptor in membrane. 10.1016/s0006-291x(02)00701-5 12150987

[30] Suzuki S, Sugawara K, Satoh Y, Toyota T (1991) Insulin stimulates the generation of two putative insulin mediators, inositol-glycan and diacylglycerol in BC3H-1 myocytes. J Biol Chem 266 13: 8115–21. 2022633

[31] Muller G, Schulz A, Wied S, Frick W (2005) Regulation of lipid raft proteins by glimepiride- and insulin-induced glycosylphosphatidylinositol-specific phospholipase C in rat adipocytes. Biochem Pharmacol 69 5: 761–80. 15710354 10.1016/j.bcp.2004.11.014

[32] Jaworek CH, Calias P, Iacobucci S, d'Alarcao M (1999) Synthesis of an inositol-containing trisaccharide related to insulin signal transduction. Tetrahedron Lett 40 4: 667–670.

[33] Hecht ML, Tsai YH, Liu X, Wolfrum C, Seeberger PH (2010) Synthetic inositol phosphoglycans related to GPI lack insulin-mimetic activity. ACS Chem Biol 5 11: 1075–1086. 20825209 10.1021/cb1002152

[34] Roberts WL, Myher JJ, Kuksis A, Low MG, Rosenberry TL (1988) Lipid analysis of the glycoinositol phospholipid membrane anchor of human erythrocyte acetylcholinesterase. Palmitoylation of inositol results in resistance to phosphatidylinositol-specific phospholipase C. J Biol Chem 263: 18766–18775. 2848806

[35] Schofield JN, Rademacher TW (2000) Structure and expression of the human glycosylphosphatidylinositol phospholipase D1 (GPLD1) gene. Biochim Biophys Acta 1494 1–2: 189–194. 11072085 10.1016/s0167-4781(00)00194-9

[36] Rhode H, Lopatta E, Schulze M, Pascual C, Schulze HP, et al (1999) Glycosylphosphatidylinositol-specific phospholipase D in blood serum: is the liver the only source of the enzyme? Clin Chim Acta 281 1–2: 127–145. 10217634 10.1016/s0009-8981(98)00218-6

[37] Deeg MA, Verchere CB (1997) Regulation of glycosylphosphatidylinositol-specific phospholipase D secretion from beta TC3 cells. Endocrinology 138 2: 819–826. 9003020 10.1210/endo.138.2.4940

[38] Metz CN, Zhang YY, Guo Y, Tsang TC, Kochan JP, et al (1991) Production of the glycosylphosphatidylinositol-specific phospholipase D by the islets of Langerhans. J Biol Chem 266 27: 17733–17736. 1833386

[39] Bowen RF, Raikwar NS, Olson LK, Deeg MA (2001) Glucose and insulin regulate glycosylphosphatidylinositol-specific phospholipase D expression in islet beta cells. Metabolism 50 12: 1489–1492. 11735099 10.1053/meta.2001.28087

[40] Schofield JN, Stephens JW, Hurel SJ, Bell KM, deSouza JB, et al (2002) Insulin reduces serum glycosylphosphatidylinositol phospholipase D levels in human type I diabetic patients and streptozotocin diabetic rats. Mol Genet Metab 75 2: 154–161. 11855934 10.1006/mgme.2001.3287

[41] Deeg MA, Bowen RF, Williams MD, Olson LK, Kirk EA, et al (2001) Increased expression of GPI-specific phospholipase D in mouse models of type 1 diabetes. Am J Physiol Endocrinol Metab 281 1: E147–54. 11404232 10.1152/ajpendo.2001.281.1.E147

[42] Ruiz-Albusac JM, Velázquez E, Iglesias J, Jimenez E, Blázquez E (1997) Insulin promotes the hydrolysis of a glycosyl phosphatidylinositol in cultured rat astroglial cells. J Neurochem 68 1: 10–19. 8978704 10.1046/j.1471-4159.1997.68010010.x

[43] Kurtz TA, Fineberg NS, Considine RV, Deeg MA (2004) Insulin resistance is associated with increased serum levels of glycosylphosphatidylinositol-specific phospholipase D. Metabolism 53 2: 138–139. 14767861 10.1016/j.metabol.2003.09.004

[44] Gray DL, O'Brien KD, D'Alessio DA, Brehm BJ, Deeg MA (2008) Plasma glycosylphosphatidylinositol-specific phospholipase D predicts the change in insulin sensitivity in response to a low-fat but not a low-carbohydrate diet in obese women. Metabolism 57 4: 473–478. 18328347 10.1016/j.metabol.2007.11.007 PMC3857163

[45] Deborde S, Schofield JN, Rademacher TW (2003) Placental GPI-PLD is of maternal origin and its GPI substrate is absent from placentae of pregnancies associated with pre-eclampsia. J Reprod Immunol 59 2: 277–294. 12896829 10.1016/s0165-0378(03)00054-8

[46] Pañeda C, Villar AV, Alonso A, Goñi FM, Varela F, et al (2001) Purification and characterization of insulin-mimetic inositol phosphoglycan-like molecules from grass pea (Lathyrus sativus) seeds. Mol Med 7 7: 454–460. 11683370 PMC1950051

[47] Deeg MA (1994) GPI-specific phospholipase D as an apolipoprotein. Braz J Med Biol Res 27 2: 375–381. 8081252

[48] Deeg MA, Bierman EL, Cheung MC (2001) GPI-specific phospholipase D associates with an apoA-I- and apoA-IV-containing complex. J Lipid Res 42 3: 442–451. 11254757

[49] Raikwar NS, Cho WK, Bowen RF, Deeg MA (2006) Glycosylphosphatidylinositol-specific phospholipase D influences triglyceride-rich lipoprotein metabolism. Am J Physiol Endocrinol Metab 290 3: E463–470. 16219662 10.1152/ajpendo.00593.2004

[50] Deeg MA, Raikwar NS, Johnson C, Williams CD (2007) Statin therapy reduces serum levels of glycosylphosphatidylinositol-specific phospholipase D. Transl Res 150 3: 153–157. 17761367 10.1016/j.trsl.2007.03.008

[51] Chalasani N, Vuppalanchi R, Raikwar NS, Deeg MA (2006) Glycosylphosphatidylinositol-specific phospholipase d in nonalcoholic Fatty liver disease: a preliminary study. J Clin Endocrinol Metab 91 6: 2279–2285. 16595594 10.1210/jc.2006-0075

[52] Armesto J, Hannappel E, Leopold K, Fischer W, Bublitz R, et al (1996) Microheterogeneity of the hydrophobic and hydrophilic part of the glycosylphosphatidylinositol anchor of alkaline phosphatase from calf intestine. Eur J Biochem 238 1: 259–69. 8665945 10.1111/j.1432-1033.1996.0259q.x

[53] Yamada T, Oka Y, Katagiri H (2008) Inter-organ metabolic communication involved in energy homeostasis: Potential therapeutic targets for obesity and metabolic syndrome. Pharmacol Ther 117: 188–198. 18006064 10.1016/j.pharmthera.2007.09.006

