# Supplementary figures and images for: Insulin-Mimicking Bioactivities of Acylated Inositol Glycans in Several Mouse Models of Diabetes with or without Obesity
Source: PLoS One. 2014 Jun 27;9(6):e100466. doi: 10.1371/journal.pone.0100466 (PMC4074071; doi:10.1371/journal.pone.0100466)

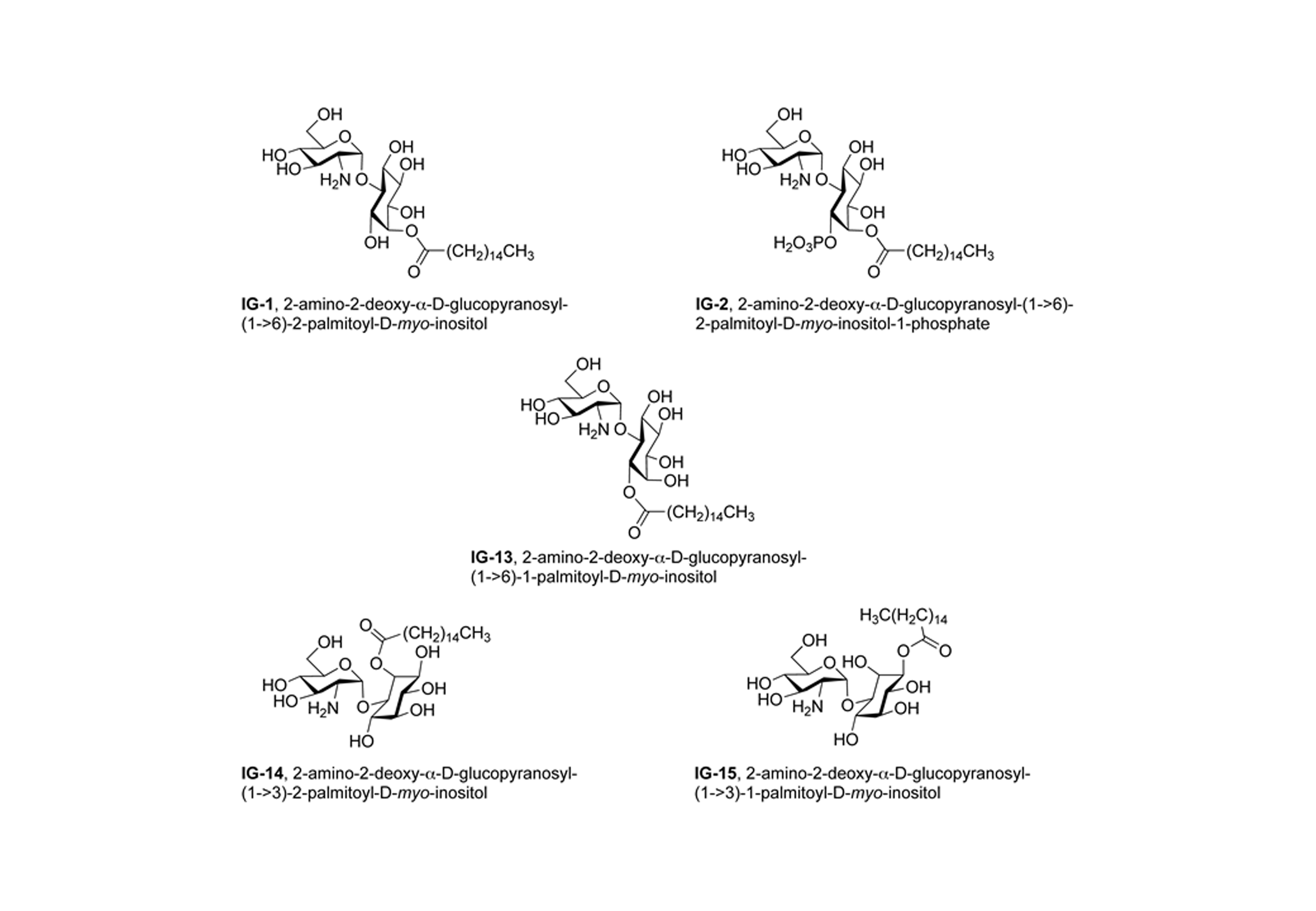

Supplement: Figure S1 — Chemical structures of synthetic acylated inositol glycans. (ZIP) [file pone.0100466.s001.zip › Figure 1S.TIF]

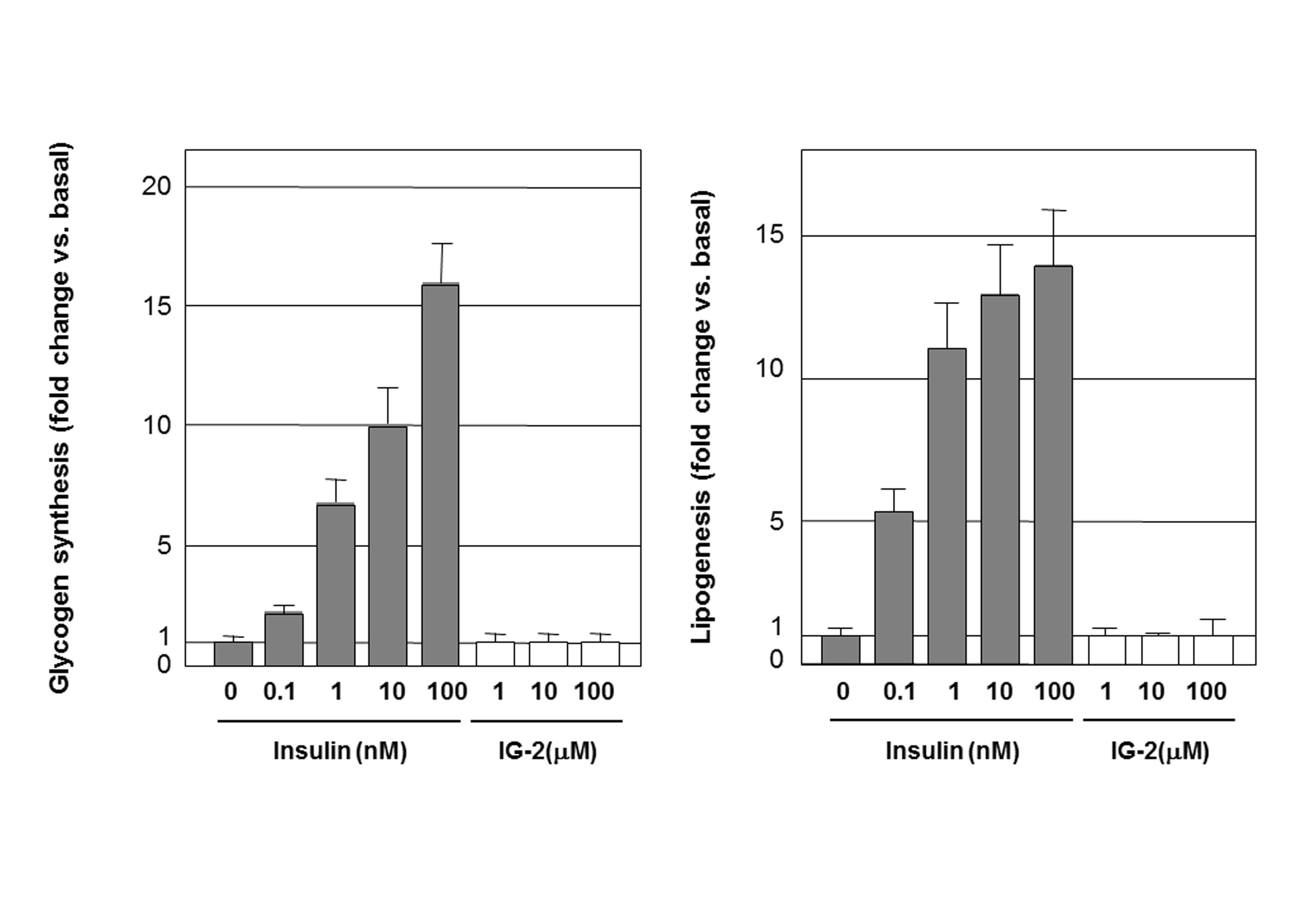

Supplement: Figure S2 — The effects of IG-1, IG-2, IG-13, IG-14 and IG-15 on lipogenesis in native rat adipocytes. A: Rat adipocytes were incubated with 6-[3H]-glucose (0.55 mM) and various concentrations of IG-1 and IG-2 for 1 hour. Incorporation of tritium into lipids was measured and is expressed as a percent of the maximal insulin response (%MIR). Red circles: IG-1 (0–60 µM); black squares: IG-2 (0–60 µM). Each data point is the average of at least five replicates. Error bars represent ±1 SD. B: Rat adipocytes were incubated with 6-[3H]-glucose (0.55 mM) and 40 µM of IG-1, IG-2, IG-13, IG-14, or IG-15 for 1 hour. Incorporation of tritium into lipids was measured and is expressed as a percent of the maximal insulin response (%MIR). Each data point is the average of at least five replicates. Error bars represent ±1 SD. (ZIP) [file pone.0100466.s002.zip › Figure 2S.TIF]

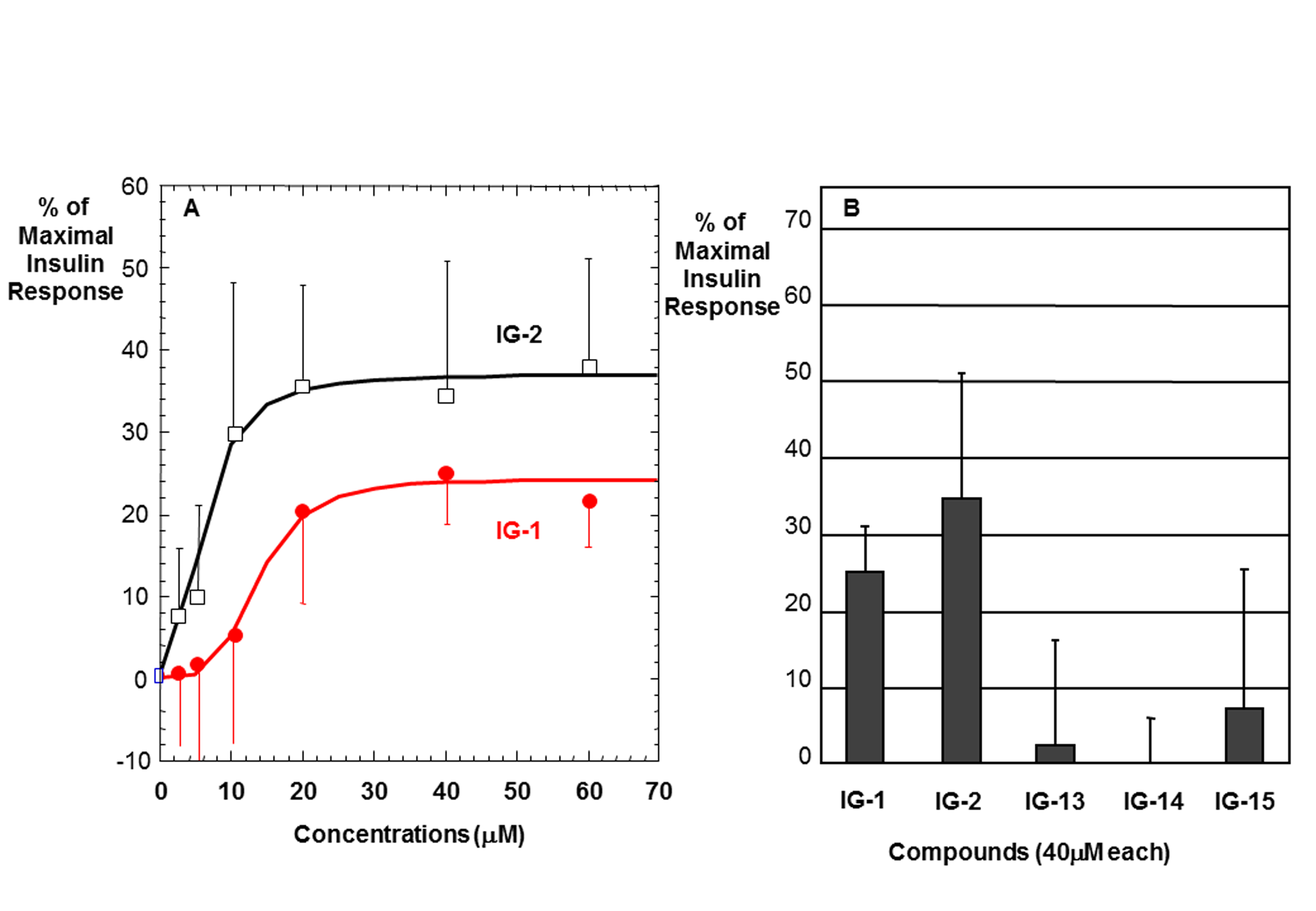

Supplement: Figure S3 — IG-2 did not stimulated glycogen synthesis and lipogenesis in 3T3-L1 adipocytes. 3T3-L1 adipocytes were treated with various concentrations of IG-2 in DMSO or 1–100 nM insulin for 30 min. Then 1 µCi of [14C]-glucose (approximately 220 cpm/nmol) was added and glycogen and lipid synthesis were measured as described in Methods and Materials. Values are expressed as fold change vs. basal. Graphs show the means±SEM of 3 independent experiments. (ZIP) [file pone.0100466.s003.zip › Figure 3S.TIF]
